# Qushi Huayu decoction ameliorates non-alcoholic fatty liver disease in rats by modulating gut microbiota and serum lipids

**DOI:** 10.3389/fendo.2023.1272214

**Published:** 2023-10-11

**Authors:** Yiming Ni, Xin Wang, Qian Wu, Yichen Yao, Yuan Xu, Yuanyuan Li, Qin Feng, Mingmei Zhou, Xiaojun Gou

**Affiliations:** ^1^ Institute for Interdisciplinary Medicine Sciences, Shanghai University of Traditional Chinese Medicine, Shanghai, China; ^2^ Central Laboratory, Baoshan District Hospital of Integrated Traditional Chinese and Western Medicine of Shanghai, Shanghai University of Traditional Chinese Medicine, Shanghai, China; ^3^ Institute of Liver Disease, Shuguang Hospital, Shanghai University of Traditional Chinese Medicine, Shanghai, China; ^4^ NHC Key Lab of Reproduction Regulation (Shanghai Institute for Biomedical and Pharmaceutical Technologies), Fudan University, Shanghai, China; ^5^ Shanghai Frontiers Science Center of TCM Chemical Biology, Institute of Interdisciplinary Integrative Medicine Research, Shanghai University of Traditional Chinese Medicine, Shanghai, China

**Keywords:** Qushi Huayu decoction, non-alcoholic fatty liver disease, lipidomics, gut microbiota, traditional Chinese medicine

## Abstract

**Introduction:**

Non-alcoholic fatty liver disease (NAFLD) is a multifactorial disease. As a clinical empirical prescription of traditional Chinese medicine, Qushi Huayu decoction (QHD) has attracted considerable attention for its advantages in multi-target treatment of NAFLD. However, the intervention mechanism of QHD on abnormal lipid levels and gut microbiota in NAFLD has not been reported.

**Methods:**

Therefore, we verified the therapeutic effect of QHD on high-fat diet (HFD)-induced NAFLD in rats by physiological parameters and histopathological examination. In addition, studies on gut microbiota and serum lipidomics based on 16S rRNA sequencing and ultra-high performance liquid chromatography-mass spectrometry (UPLC-MS) were conducted to elucidate the therapeutic mechanism of NAFLD in QHD.

**Results:**

The changes in gut microbiota in NAFLD rats are mainly reflected in their diversity and composition, while QHD treated rats restored these changes. The genera *Blautia, Lactobacillus, Allobaculum, Lachnoclostridium* and *Bacteroides* were predominant in the NAFLD group, whereas, *Turicibacter, Blautia, Sporosarcina, Romboutsia, Clostridium_sensu_stricto_1, Allobaculum*, and *Psychrobacter* were predominant in the NAFLD+QHD group. Lipid subclasses, including diacylglycerol (DG), triglycerides (TG), phosphatidylethanolamine (PE), phosphatidylcholine (PC), phosphatidic acid (PA), phosphatidylserine (PS), lysophosphatidylinositol (LPI), and phosphatidylglycerol (PG), were significantly different between the NAFLD and the control groups, while QHD treatment significantly altered the levels of DG, TG, PA, lysophosphatidylcholine (LPC), lysophosphatidylethanolamine (LPE), and platelet activating factor (PAF). Finally, Spearman’s correlation analysis showed that NAFLD related differential lipid molecules were mainly associated with the genera of *Bacteroides, Blautia, Lachnoclostridium, Clostridium_sensu_stricto_1*, and *Turicibacter*, which were also significantly correlated with the biological parameters of NAFLD.

**Discussion:**

Taken together, QHD may exert beneficial effects by regulating the gut microbiota and thus intervening in serum lipids.

## 1 Introduction

Non-alcoholic fatty liver disease (NAFLD), one of the most significant forms of chronic liver disease worldwide, is characterized by hepatic steatosis. Non-alcoholic steatohepatitis is the advanced form of NAFLD, and a certain proportion of patients will progress to cirrhosis and hepatocellular carcinoma (1–4). The global prevalence of NAFLD is 32.4%, and the incidence rate of NAFLD will continue to increase in the coming years due to the obesity pandemic (5, 6). Strategies to raise awareness and address all aspects of NAFLD are urgently needed.

Traditional Chinese medicine is widely used as an alternative medicine and shows remarkable efficacy in NAFLD due to its multi-target advantages (7, 8). However, each coin has two sides. Due to the complex components and interactions in the Chinese herbs, it is difficult to elucidate the mechanism of prescriptions containing several herbs. As a clinical empirical prescription, Qushi Huayu decoction (QHD) has been used in the intervention of NAFLD in China for several decades (9, 10). The efficacy of QHD has been demonstrated in several models of NAFLD *in vivo* and *in vitro* (9, 11–13), and the mechanisms include regulation of branched-chain amino acid metabolism disorder, upregulation of AMPK/SIRT1/UCP-1 signaling pathway (14), modulation of fatty acid β-oxidation (15), regulation of gut microbiota composition, and protection of intestinal tight junctions (16).

Altered gut microbiota is closely associated with the pathogenesis of several metabolic diseases, including obesity, type 2 diabetes and NAFLD (17–19). In particular, dysbiosis of gut microbiota and its metabolites promotes the development of NAFLD through multiple mechanisms, including disruption of lipid metabolism in the liver, promoting fat accumulation and lipotoxicity (20).

Under normal physiological conditions, lipids exist in the form of aggregates within the membrane and perform various functions. In addition to being components of biological membranes, lipids are also involved in energy metabolism and storage and can play an important role as signaling molecules (21). Accumulating research has demonstrated that NAFLD is typically accompanied by excessive specific lipids, such as free fatty acid, ceramides, free cholesterol, and bile acids, not just triglycerides (TG, a traditional clinical biochemical marker of NAFLD). These excess lipids may cause liver toxicity through various mechanisms, including JNK and death receptors (22), endoplasmic reticulum stress, mitochondrial dysfunction, and oxidative stress, leading to hepatocyte damage and inflammation. This condition, known as lipotoxicity, may cause the progression of NAFLD to NASH (20). Although lipids have important pathological significance for NAFLD, they are currently one of the least studied cellular biomolecules (23). The recent rise of high-throughput measurement based lipidomics is an emerging large-scale study of lipid metabolites, revealing disordered pathways and lipid biomarkers that can be used to demonstrate therapeutic efficacy (24–26). Through this advanced technology, the pathological mechanism of NAFLD can be understood on a broader lipid profiling and the efficacy of drugs can be evaluated.

Therefore, 16S rRNA sequencing and serum lipidomics based on ultra-high performance liquid chromatography-mass spectrometry (UPLC-MS)-analysis were performed to investigate the effect of QHD in NAFLD intervention, screen for dominant gut genera and serum lipid metabolites, and evaluate the relationship between gut microbiota and serum lipid metabolites.

## 2 Materials and methods

### 2.1 Preparation of QHD

The method of preparation was according to an earlier report (16). *Artemisiae scopariae herba* (“Yinchen” in Chinese), *Polygoni cuspinati rhizome et radix* (“Huzhang” in Chinese), *Herba hyperici japonica* (“Tianjihuang” in Chinese), *Curcumae longae rhizome* (“Jianghuang” in Chinese) and *Gardeniae fructus* (“Zhizi” in Chinese) were prepared in the ratio of 6: 4: 4: 3: 3. The concentration for the final stock solution of QHD extract was adjusted to 0.93 g/crude herb/mL.

### 2.2 Animal treatment

Wistar rats were obtained from the Shanghai Experimental Animal Center of Chinese Academy of Sciences. All experimental procedures were approved by the Animal Studies Ethics Committee of Shanghai University of Traditional Chinese Medicine (Ethical number: PZSHUTCM201127007). A total of 24 male Wistar rats weighing 160~180 g were kept at 23 ± 3°C with a 12h light/dark cycle. After one week of acclimation, we randomly divided the rats into the control, NAFLD, and NAFLD+QHD groups (n=8 per group). A standard diet was given to control rats for 8 weeks, while those in the NAFLD and NAFLD+QHD groups were NAFLD-induced by feeding high-fat diet (HFD) containing 2% cholesterol and 10% lard (No. FB-M10L2CH) for 8 weeks. Rats in the NAFLD+QHD group were administrated 0.1 mL/kg QHD by gavage for the last 4 weeks. The dosage was determined by the results of previous studies on QHD in NAFLD rats (9, 12). The control rats and NAFLD rats received equal sterile water.

### 2.3 Physiological parameters measurements

After an overnight fast of 12 h, fasting blood glucose (FBG) was measured with a glucometer (Yuwell, model 740, Jiangsu, China). After anesthesia, blood was collected from the abdominal aorta, and serum was separated by centrifugation (1000 × g, 4°C, 15 min). Serum insulin was detected using ELISA kits (Crystal Chem). Homeostasis model assessment of insulin resistance (HOMA-IR) was performed: HOMA-IR = FBG (mmol/L) × insulin (mU/L)/22.5). An alanine aminotransferase (ALT) biochemistry assay kit came from the Nanjing Jiancheng Institute of Biotechnology was used to examine serum ALT according to the manufacturer’s instructions.

After excision, the livers were weighed. Some of the tissues were fixed with formalin for histopathological observation, and the rest were immediately frozen for further analysis. Liver TG was determined using a TG assay kit purchased from Dongou Biology Technique Co. Ltd. (Zhejiang, China) according to the manufacturer’s instructions.

### 2.4 Histological analyses

Lipids of liver tissues from different groups were observed by staining with oil red O dye. Liver tissues were stored in tubes and snap frozen in liquid nitrogen. The 10 μm thick liver sections were stained with oil red to visualize the lipid accumulation within the hepatocyte. To observe the pathological changes, the liver tissues were fixed in 4% paraformaldehyde and embedded in paraffin. The 3 μm thick liver sections were stained with hematoxylin and eosin (HE) and examined under a light microscope. The NAFLD activity score (NAS) was evaluated with a higher score indicating increasing severity according to the following features: hepatocyte steatosis (< 5% = 0; 5~33% = 1; 33~66% = 2; >66% = 3); lobular inflammation (Necrotic foci were counted at 20x) (none = 0; < 2 = 1; 2~4 foci = 2; > 4 foci = 3); and hepatocellular ballooning (none = 0; few = 1; prominent = 2) (27).

### 2.5 16S rRNA sequencing

After 8 weeks of experiment, rats are individually housed in a metabolic cage, and feces and urine are immediately dropped into the containers below separately. During the collection process, we need to ensure that the feces are fresh and the operation is aseptic to obtain subsequent reliable gut microbiota analysis data. As the metabolic cages were used, feces were collected separately in a collection bottle, and we obtained the feces samples from the bottle. Feces samples of each rat were collected separately and stored in -80°C freezers until processing. We applied the OMEGA Soil DNA Kit (M5635-02) (Omega Bio-Tek, Norcross, GA, USA) to extract total bacterial DNA. We used a NanoDrop NC2000 spectrophotometer and agarose gel electrophoresis to evaluate the extracted DNAs (**Supplementary Table S1**). The V3-V4 regions of 16S rRNA were identified by primers 338F (5’-ACTCCTACGGGAGGCAGCA-3’) and 806R (5’-GGACTACHVGGGTWTCTAAT-3’). The Illumina NovaSeq platform with NovaSeq 6000 SP Reagent Kit (500 cycles) was used to perform the sequencing.

The bioinformatics analysis of the microbiome was performed using QIIME2 2019.4. The demux plugin was used to demultiplex the raw sequence data, followed by primer cutting with the cutadapt plugin. DADA2 plugin was then utilized for quality filtering, denoising, merging and chimera removal. Operational taxonomic units (OTUs) were compared using MAFFT, and FastTree 2 was utilized to construct a phylogeny. The diversity plugin was used to estimate alpha and beta diversity metrics. Phylogenetic Investigation of Communities by Reconstruction of Unobserved States 2 (PICRUSt2) analysis based on KEGG pathway was performed to predict the function of the gut microbiota.

### 2.6 Lipidomics analysis

#### 2.6.1 Lipids extraction

Blood was collected from rats and allowed to stand at room temperature before serum was obtained by centrifugation (1000 × g, 4°C, 15 min). Briefly, 50 μL of serum was collected; 1.5 mL of dichloromethane/methanol (2/1, vol/vol) and two internal standards, LPC (12:0) and PC (11:0/11:0), were added; and the homogenate was centrifuged (3,000 rpm, 15 min). The bottom layer of the resulting liquid was moved into a new centrifuge tube, adding 1.5 mL dichloromethane/methanol (2/1, vol/vol), and the top layer of the liquid was treated to obtain the homogenate. Two resulting liquids were mixed and dried in a freeze-concentration centrifugal dryer. Then we dissolved the powder in isopropanol/methanol (1/1, vol/vol) and saved it at minus 20°C for lipidome analysis.

#### 2.6.2 UPLC-MS analysis

Lipidome analysis was performed on an Ultimate™ 3000 UPLC coupled a Q Exactive hybrid quadrupole-Orbitrap Mass Spectrometers system (Thermo Fisher) with a hypersil GOLD C18 column (100×2.1 mm, 1.9 μm). The temperature of column was set at 45°C. Eluents A was 10 mmol/L ammonium formate in 40% water and 60% acetonitrile; and eluent B was 10 mmol/L ammonium formate in 90% isopropanol and 10% acetonitrile. We set the flow rate at 0.35 mL/min, injected ten microliters, and set the gradient at 30~100% B in 14.5 min, 100% B in 14.5~16.5 min, 100~30% B in 16.50~16.51 min, and 30% B in 16.51~20 min.

The spray voltages of MS were at 3.0 kV in the ESI+ mode and at 2.8 kV in the ESI- mode. The capillary temperature was set at 350°C, with a sheath gas flow rate of 35 arb, an aux gas flow rate of 15 arb, and a sweep gas flow rate at one arb. The heater temperature was maintained at 350°C. The S-Lens RF level was 50. MS operation was performed at a resolving power of 70,000 in full scan mode (ESI+, 250~1500 m/z; ESI-, 200~1500m/z).

#### 2.6.3 Primary data processing

First, the raw data were received by performing Xcalibur (version 3.0), and LipidSearch (version 4.0) was operated to identify and quantify the lipids. The identification was based on the retention time, exact mass, and pattern of precursor ions and MS2. The key procedure included a precursor tolerance of 5 ppm, a product tolerance of 10 ppm, an intensity threshold of product ion of 5%, an m-score threshold of 3.0, a quantitation m/z tolerance of ±5.0 ppm, and a quantification RT range of ±1.0 min. The main mode filter was main isomer peak and ID quality filters A, B, C, and D; and the adduct ions were: +H, +NH4, and +Na for ESI+ mode, and −H, +HCOO, and +CH3COO for ESI- mode. Alignment parameters were LC-MS data within 0.25 min RT time window; m-score threshold: 3.0; c-score threshold: 2.0; all isomer peak; ID quality filters A, B, C and D. Data acquired from each experimental rat constructed a raw data matrix, including the sample information, the identified lipids, lipid classification, retention time, charge-mass ratio, and peak area.

#### 2.6.4 Lipids analysis

Lipid analyses were performed on the lipid metabolites using SIMCA software (version 14.1) and SPSS (version 21.0). Metabolic enrichment of differential lipids was performed using MetaboAnalyst 5.0 (https://www.metaboanalyst.ca).

### 2.7 Statistical analysis

The software GraphPad Prism (version 9.0.0) was used for statistical analysis. All data are shown as the mean ± standard deviation. We used the Shapiro-Wilk normality test to verify normality. One-way ANOVA was utilized to examine the normally distributed data among the groups. The data that did not satisfy normal distribution were examined using the Kruskal-Wallis test. Differences with statistical significance were marked by: * *P* < 0.05, ** *P* < 0.01, *** *P* < 0.001, and **** *P* < 0.0001.

## 3 Results

### 3.1 QHD attenuated NAFLD-induced insulin resistance and hepatic changes

**Figure 1:**
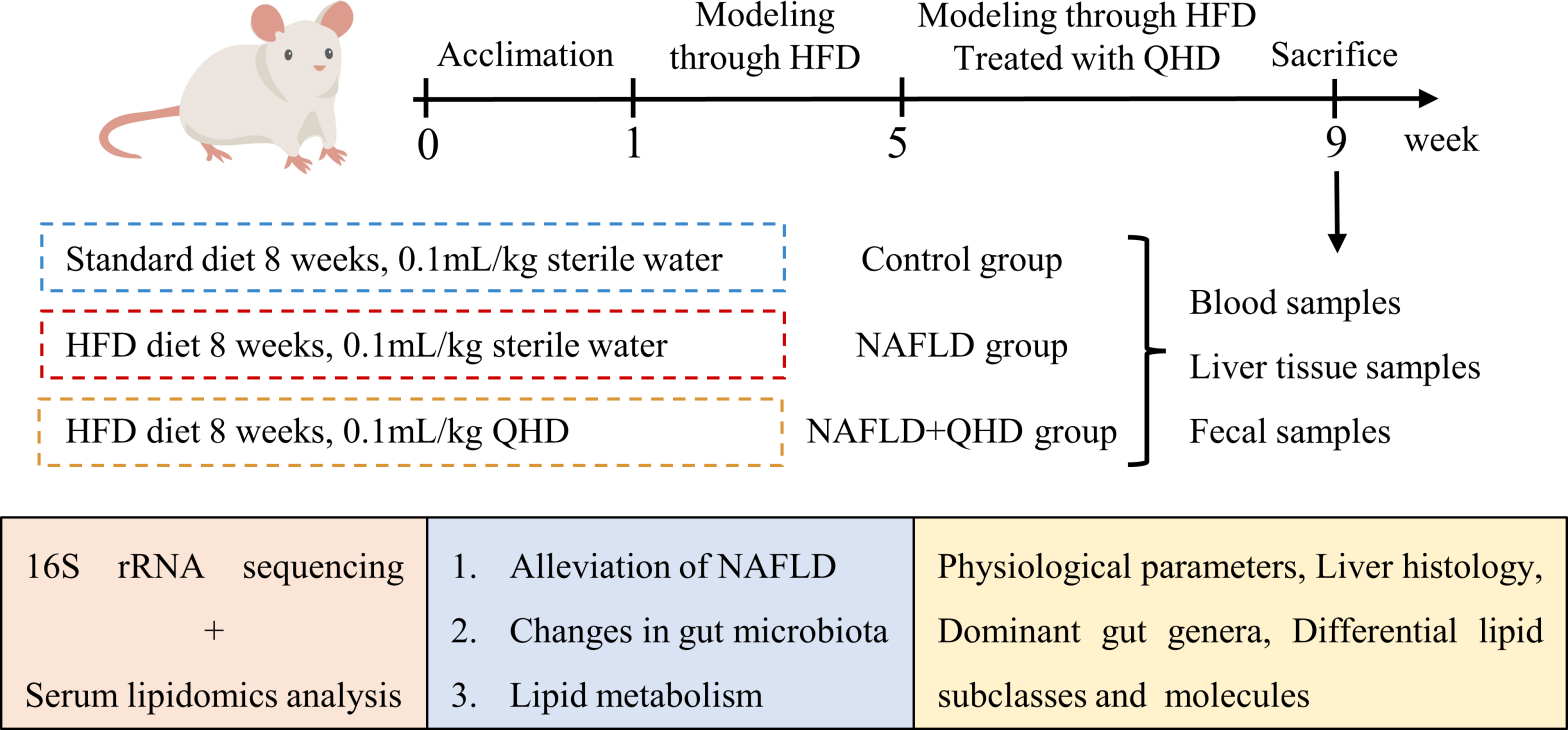
Schematic illustration showing the design of the experiment.

**Figure 2:**
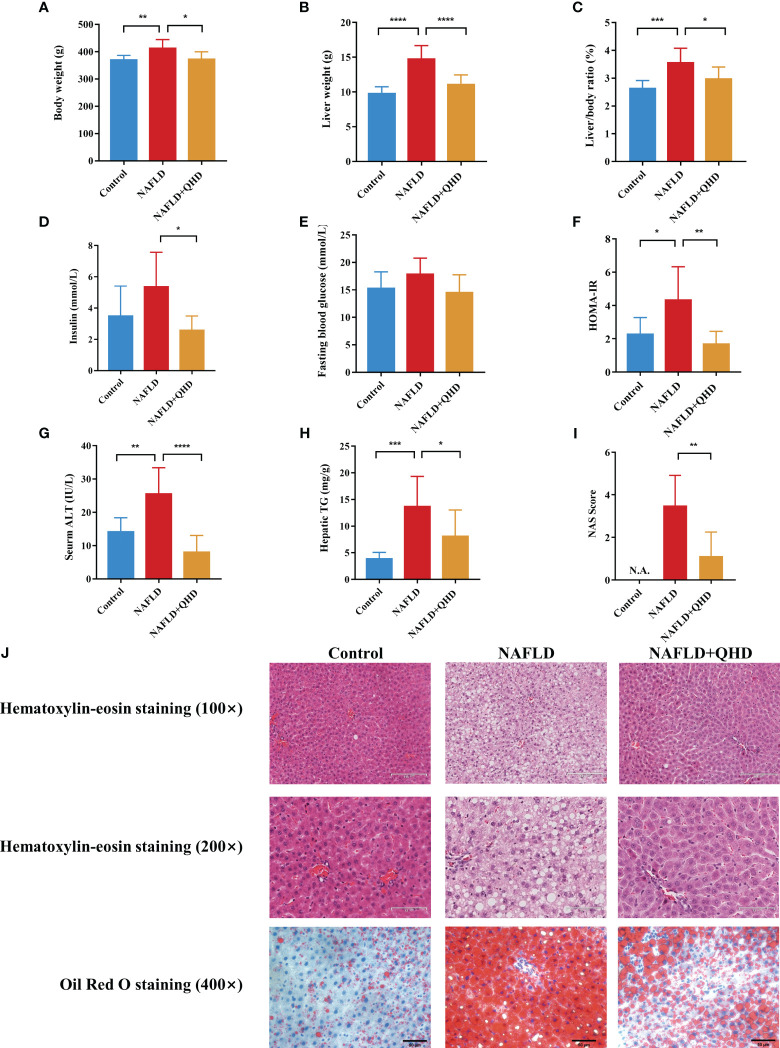
Evaluation of the therapeutic effects of QHD on NAFLD rats (n=8). **(A)** Body weight; **(B)** liver weight; **(C)** liver/body ratio; **(D)** insulin; **(E)** fasting blood glucose; **(F)** HOMA-IR; **(G)** serum ALT; **(H)** hepatic TG; **(I)** NAS score. **(J)** Representative pictures of HE staining (100× and 200× original magnification) and oil red O staining (400× original magnification) of rats’ liver. Statistical significance was considered at * *P* < 0.05, ** *P* < 0.01, *** *P* < 0.001, and **** *P* < 0.0001. QHD, Qushi Huayu decoction; NAFLD, non-alcoholic fatty liver disease; HOMA-IR, homeostasis model assessment of insulin resistance; ALT, alanine aminotransferase; HE, hematoxylin-eosin; NAS, NAFLD activity score.

To investigate whether QHD can attenuated NAFLD, the rats were randomly assigned into 3 groups, control, NAFLD, and NAFLD+QHD groups (n=8 per group, **Figure 1**). After HFD feeding, NAFLD rats showed significantly increased body weight (*P* = 0.0056, **Figure 2A**), liver weight (*P* < 0.0001, **Figure 2B**), liver/body ratio (*P* = 0.0003, **Figure 2C**), HOMA-IR index (*P* = 0.0135, **Figure 2F**), serum ALT (*P* = 0.0018, **Figure 2G**), and hepatic TG (*P* = 0.0005, **Figure 2H**) compared with rats in the Control group. Compared with the NAFLD group, the body weight (*P* = 0.0194), liver weight (*P* < 0.0001) and liver/body ratio (*P* = 0.0197) of the NAFLD+QHD rats were significantly reduced. QHD treatment also reduced insulin (*P* = 0.0108, **Figure 2D**) and improved HOMA-IR (*P* = 0.0018) in NAFLD rats. However, FBG did not differ significantly (*P* > 0.05, **Figure 2E**). Apparently, QHD restored liver function and TG accumulation in the liver, as indicated by decreased serum ALT (*P* < 0.0001) and hepatic TG (*P* = 0.0409), respectively.

In HE-stained histopathologic images, liver samples from the NAFLD group showed hepatocyte degeneration, inflammatory infiltration, abundant steatosis, and prominent hepatocyte ballooning. These histopathologic features were alleviated in the NAFLD+QHD group (**Figure 2J**). NAS score was relatively lower in NAFLD+QHD group than NAFLD (*P* = 0.0023, **Figure 2I**). The histopathological image with oil red O staining showed prominent hepatic steatosis, large lipid droplets, a darkly stained central lobular area, and a lightly stained marginal area in NAFLD rats. The above pathological changes were significantly reduced after QHD treatment. These outcomes implied that the NAFLD model induced by HFD was successful, and the histopathological changes were ameliorated after QHD administration.

### 3.2 QHD altered gut microbiota dysbiosis in NAFLD rats

**Figure 3:**
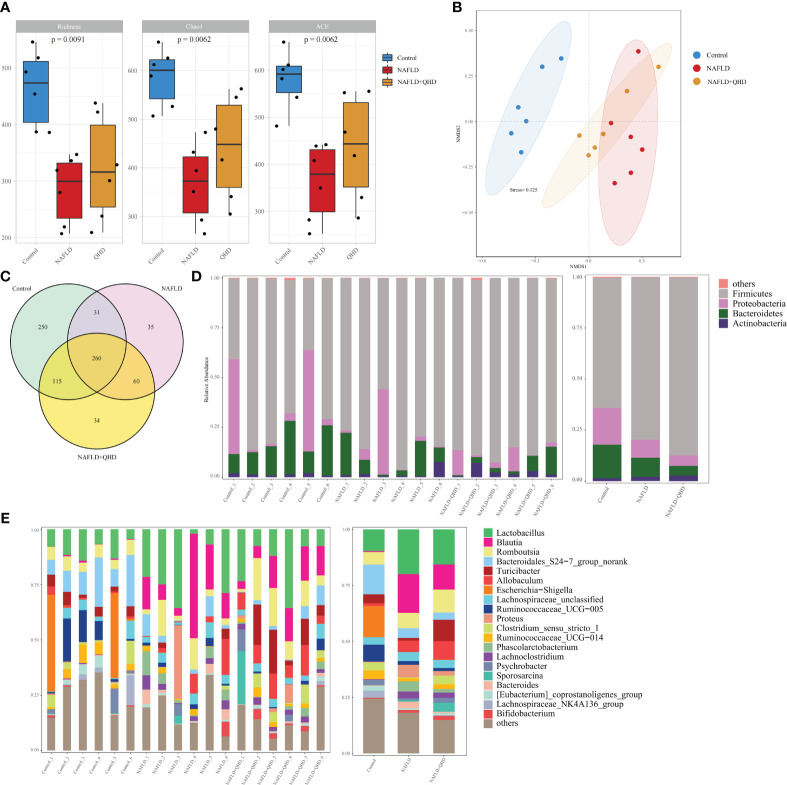
QHD altered gut microbiota diversity and composition in NAFLD rats (n=6). **(A)** Gut microbiota α-diversity; **(B)** NMDS analysis; **(C)** Venn diagram showed the overlap of OTUs. Barplot showing relative abundance of gut microbiota in **(D)** phylum and **(E)** genus levels. NAFLD, non-alcoholic fatty liver disease; QHD, Qushi Huayu decoction; NMDS, nonmetric multidimensional scaling.

Increasing evidence confirmed that gut microbiota is vital in developing NAFLD (5, 28–31). We conducted 16S rRNA sequencing of fecal samples to study the influence of QHD on gut microbiota. HFD induced lower diversity and richness of gut microbiota, as proved by the reduction of richness, ace and Chao1 indexes (**Figure 3A**). However, QHD supplementation restored these indexes caused by HFD, indicating that QHD increased gut microbiota alpha-diversity. Besides, β-diversity was set to generate a nonmetric multidimensional scaling (NMDS) analysis at the OUT level. As shown in **Figure 3B**, the HFD group was separated from the other two groups, indicating gut microbiota dysbiosis caused by HFD. Venn diagram displayed the unique and shared OUT among three groups (**Figure 3C**). There were 656 OTUs and 250 unique OTUs in control rats, 386 OTUs and 35 unique OTUs in NAFLD rats, and 439 OTUs and 34 unique OTUs in NAFLD+QHD rats.

Cluster histograms displayed the taxonomy abundance of gut microbiota in phylum and genus levels in each sample and each group (**Figures 3D, E**). At the phylum level, *Firmicutes* and *Proteobacteria* dominated the gut microbiota in the three groups. The abundances of *Firmicutes* rose, while *Proteobacteria* and *Bacteroidetes* declined in NAFLD and NAFLD+QHD rats. At the genus level, *Lactobacillus, Allobaculum, Blautia, Proteus*, *Phascolarctobacterium*, *Lachnoclostridium* and *Bacteroides* were particularly increased in the gut microbiota of feces of NAFLD rats than those of the control group, whereas *Bacteroidales_S24-7_group_norank, Turicibacter, Escherichia-Shigella, Ruminococcaceae_UCG-005, Ruminococcaceae_UCG-014, Lachnospiraceae_NK4A136_group*, and *Clostridium_sensu_stricto_1* decreased in NAFLD rats. Meanwhile, gut microbiota in NAFLD+QHD rats is changing as illustrated by the decrease in *Lactobacillus, Blautia, Bacteroidales_S24-7_group_norank, Proteus, Phascolarctobacterium*, and *Bacteroides*, while *Romboutsia, Turicibacter, Allobaculum, Clostridium_sensu_stricto_1, Psychrobacter, Sporosarcina* and *Bifidobacterium* increased significantly in the gut microbiota of NAFLD+QHD rats.

**Figure 4:**
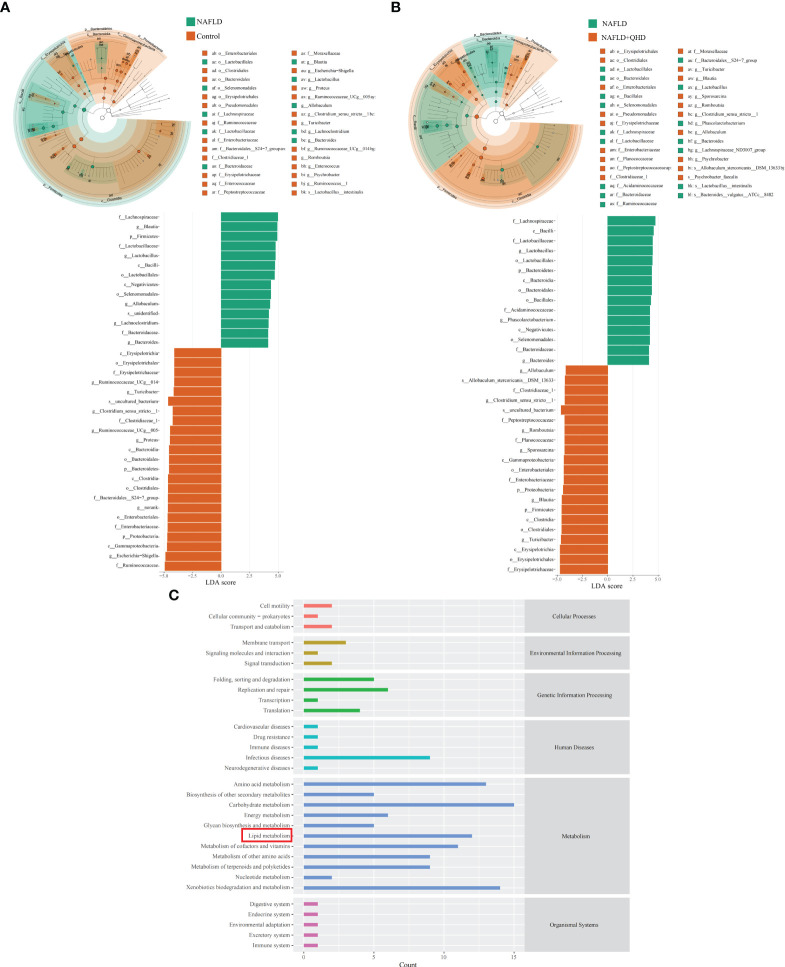
Dominant gut microbiota and function prediction (n=6). Taxonomic cladograms and linear discriminant analysis scores from LefSe analysis of **(A)** Comparison between NAFLD and control groups; **(B)** Comparison between NAFLD and NAFLD+QHD groups are shown. **(C)** PICRUSt2 analysis based on KEGG pathway was performed to predict the function of the gut microbiota. NAFLD, non-alcoholic fatty liver disease; QHD, Qushi Huayu decoction; LEfSe, linear discriminant analysis of effect size.

Linear discriminant analysis of effect size (LEfSe) analysis was performed to analyze predominant gut microbiota in the rats (**Figures 4A, B**). From the results, it could be concluded that compared to the control group, genera *Blautia, Lactobacillus, Allobaculum, Lachnoclostridium* and *Bacteroides* were predominant in the NAFLD, whereas *Turicibacter, Blautia, Sporosarcina, Romboutsia, Clostridium_sensu_stricto:1, Allobaculum*, and *Psychrobacter* were predominant in the NAFLD+QHD group compared with the NAFLD group.

To predict the function of the gut microbiota, we performed PICRUSt2 based on KEGG database and presented the average abundance of all samples by a bar graph (**Figure 4C**), which showed that the gut microbiota is largely involved in lipid metabolism.

### 3.3 QHD reversed lipid metabolomics in NAFLD rats

**Figure 5:**
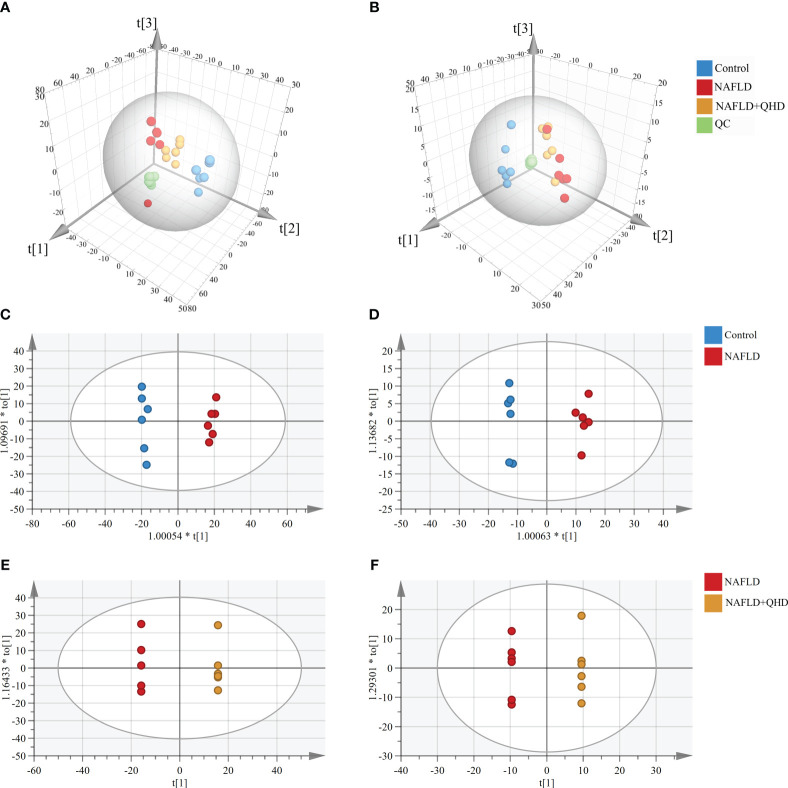
Multivariate analysis of circulating lipid profile (n=6). Three-dimensional PCA plots showing the serum lipid metabolites **(A)** in the positive ion mode and **(B)** in the negative ion mode. OPLS-DA plots of serum lipid metabolites profiling of the control and NAFLD rats **(C)** in the positive ion mode and **(D)** in the negative ion mode. OPLS-DA plots of lipid metabolites profiling of the NAFLD and NAFLD+QHD rats **(E)** in the positive ion mode and **(F)** in the negative ion mode. NAFLD, non-alcoholic fatty liver disease; QHD, Qushi Huayu decoction; PCA, principal component analyze; OPLS-DA, orthogonal projections to latent structures-discriminant analysis.

There is little knowledge about how lipids modulate NAFLD in rats. Therefore, UPLC-MS-based lipidomics was used to comprehensively characterize the lipid response upon NAFLD modeling and QHD treatment. Three-dimensional PCA plots of lipids obtained in positive (R^2^X=0.719, Q^2^ = 0.539, **Figure 5A**) and negative (R^2^X=0.652, Q^2^ = 0.533, **Figure 5B**) ion modes of serum samples indicated an obvious divergence of circulating lipid metabolites in NAFLD rats from the other two groups, indicating that the circulating lipid profile was altered by HFD and QHD administration could reverse lipid metabolomics. The orthogonal projections to latent structures-discriminant analysis (OPLS-DA) scoring plots showed each pair of comparison, Control *vs*. NAFLD in positive ion modes (R^2^X=0.545, R^2^Y=0.994, Q^2^ = 0.942, **Figure 5C**), NAFLD *vs*. NAFLD+QHD in positive ion modes (R^2^X=0.781, R^2^Y=1, Q^2^ = 0.91, **Figure 5E**), Control *vs*. NAFLD in negative ion modes (R^2^X=0.553, R^2^Y=0.993, Q^2^ = 0.944, **Figure 5D**) and NAFLD *vs*. NAFLD+QHD in negative ion modes (R^2^X=0.776, R^2^Y=1, Q^2^ = 0.802, **Figure 5F**) were separated clearly in the model.

### 3.4 Lipid subclasses and molecules differed after QHD treatment

**Figure 6:**
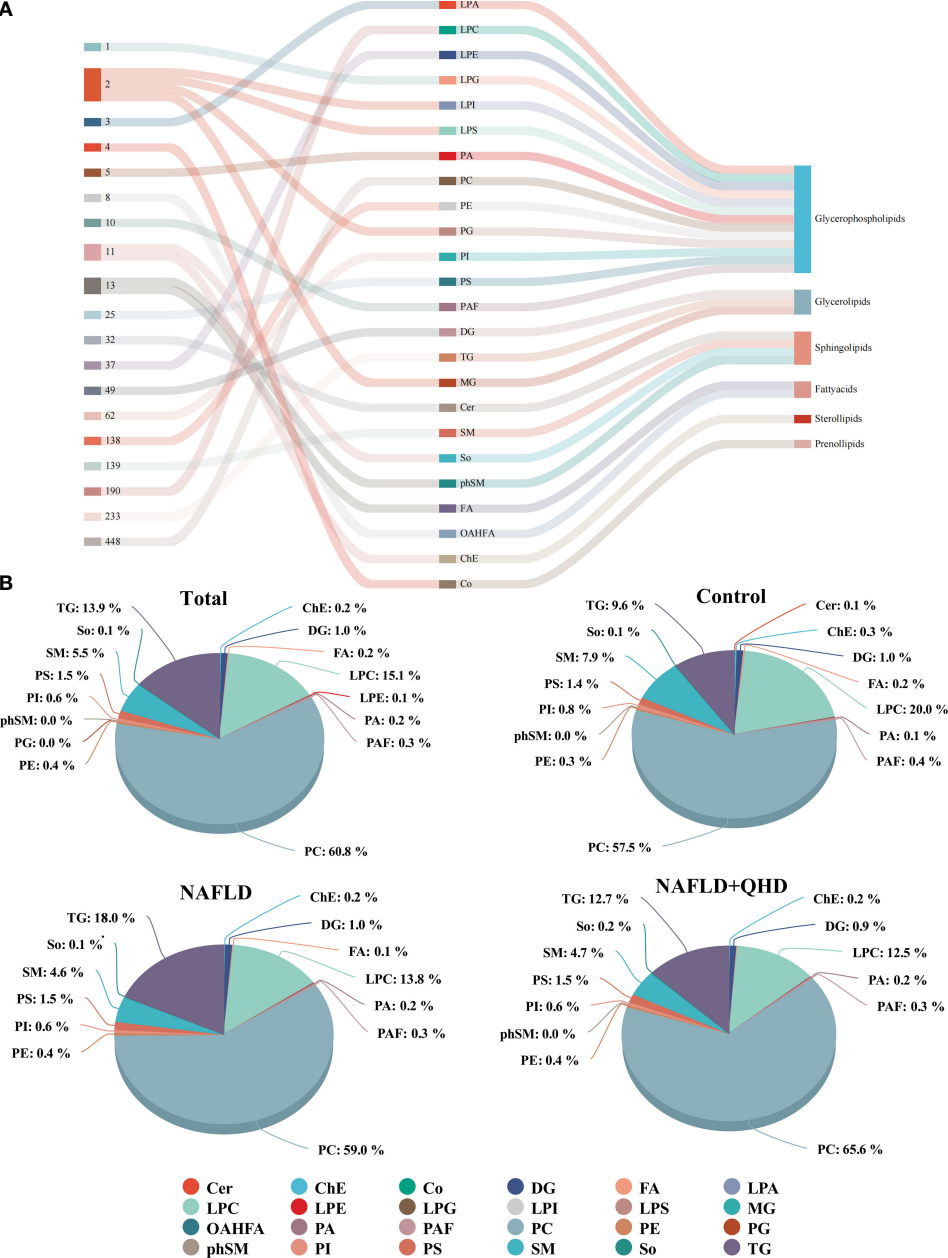
Classification and proportion of circulating lipids in rats. **(A)** Sankey diagram showing the lipid molecules numbers of each subclass and the classification; **(B)** Pie chart plot representing the proportion of each lipid subclass (expressed as a percentage of the total lipids). Cer, ceramide; ChE, cholesteryl ester; Co, coenzyme; DG, diacylglycerol; FA, fatty acid; LPA, lysophosphatidic acid; LPC, lysophosphatidylcholine; LPE, lysophosphatidylethanolamines; LPG, lysophosphoglycerol; LPI, lysophosphatidylinositol; LPS, lysophosphatidylserine; MG, monoglyceride; OAHFA, (o-acyl)-1-hydroxy fatty acid; PA, phosphatidic acid; PAF, platelet-activating factor; PC, phosphatidylcholine; PE, phosphatidylethanolamine; PG, phosphatidylglycerol; phSM, phytosphingosine; PI, phosphatidylinositol; PS, phosphatidylserine; SM, sphingomyelin; So, sphingosine; TG, triglycerides; NAFLD, non-alcoholic fatty liver disease; QHD, Qushi Huayu decoction.

**Figure 7:**
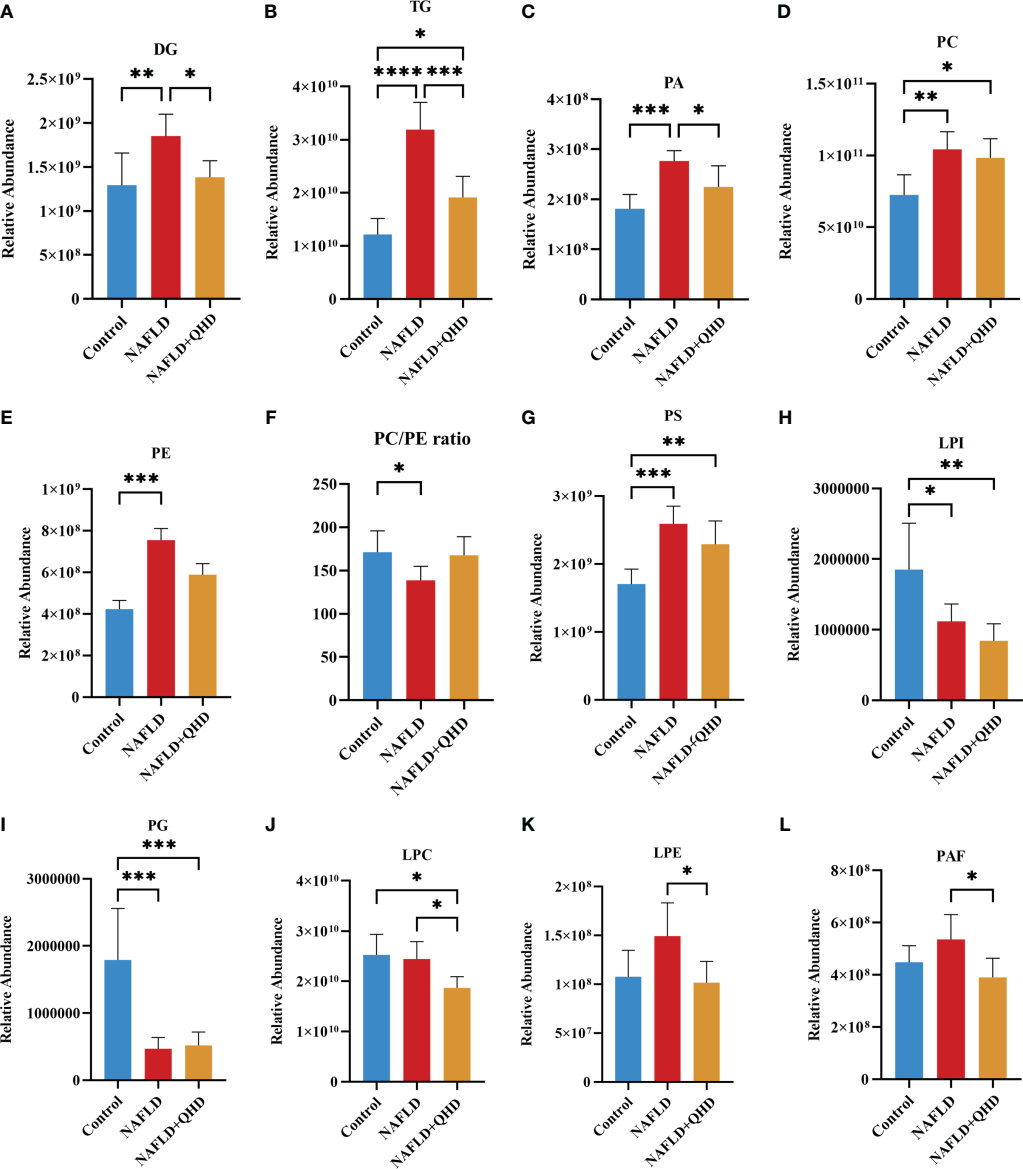
**(A–L)** Significant changes in circulating lipid subclasses (n=6). Statistical significance was considered at * *P* < 0.05, ** *P* < 0.01, *** *P* < 0.001, and **** *P* < 0.0001. DG, diacylglycerol; TG, triglycerides; PA, phosphatidic acid; PC, phosphatidylcholine; PE, phosphatidylethanolamine; PS, phosphatidylserine; LPI, lysophosphatidylinositol; PG, phosphatidylglycerol; LPC, lysophosphatidylcholine; LPE, lysophosphatidylethanolamines; PAF, platelet-activating factor; NAFLD, non-alcoholic fatty liver disease; QHD, Qushi Huayu decoction.

**Figure 8:**
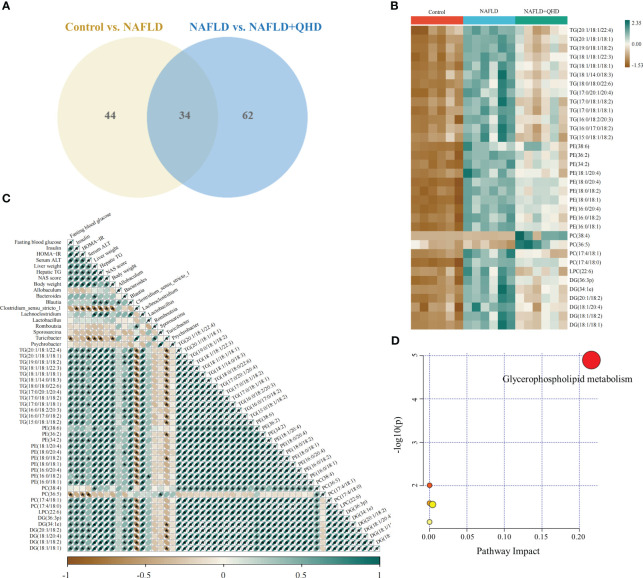
Differential lipid screening and Spearman’s correlation analysis. The projection value VIP > 1.5 in OPLS-DA and *P* < 0.05 of Students t-test were selected as potential biomarkers for further analysis (n=6). **(A)** The Venn diagram showing the overlap of differential lipid metabolites between the control and NAFLD groups and between the NAFLD and NAFLD+QHD groups; **(B)** Heatmap of the 34 differential lipids; **(C)** Heatmap showing Spearman’s correlation analysis among biological parameters, major gut genera, and lipid metabolites, with significance was indicated at * *P* < 0.05. **(D)** Lipid metabolic pathway analysis based on differential lipids in serum. VIP, variable importance in projection; OPLS-DA, orthogonal projections to latent structures-discriminant analysis; NAFLD, non-alcoholic fatty liver disease; QHD, Qushi Huayu decoction.

The lipids in serum consisted of six lipid classes, including glycerophospholipids, sphingolipids, glycerolipids, sterol lipids, FAs, and prenol lipids. The lipid molecules were examined in the positive and negative ionization modes. 1024 lipids from 17 subclasses were detected in positive ionization mode, including ceramide (Cer), cholesteryl ester (ChE), coenzyme (Co), diacylglycerol (DG), lysophosphatidylcholine (LPC), lysophosphatidylethanolamines (LPE), lysophosphatidylserine (LPS), monoglyceride (MG), phosphatidylcholine (PC), phosphatidylethanolamine (PE), phosphatidylglycerol (PG), phytosphingosine (phSM), phosphatidylinositol (PI), phosphatidylserine (PS), sphingosine (So), sphingomyelin (SM), and TG. In negative ion mode, 418 lipid species from 18 subclasses were found, including Cer, FA, lysophosphatidic acid (LPA), LPC, LPE, lysophosphoglycerol (LPG), lysophosphatidylinositol (LPI), LPS, (o-acyl)-1-hydroxy fatty acid (OAHFA), phosphatidic acid (PA), platelet-activating factor (PAF), PC, PE, PG, phSM, PI, PS, and SM. In **Figure 6A**, the Sankey diagram showed the classification of main lipid classes and the detected number of lipid molecules of each subclass. As shown by the pie charts, we analyzed the categories of lipids and summed the peak areas of each type of lipid molecule to exhibit the variation trend of each lipid subclass (**Figure 6B**). Among these subclasses, PC was the most abundant (60.8%), followed by LPC (15.1%) and TG (13.9%). In addition, the content of each subclass of lipid was compared. Remarkably, the abundance of DG, TG, PA, PC, PE and PS increased notably, while LPI and PG decreased significantly in NAFLD rats. In addition, the levels of DG, TG, LPC, LPE, PA and PAF decreased after QHD administration (**Figure 7**). 34 differential lipids were screened between the comparisons according to: variable importance in projection (VIP) > 1.5 and *P* < 0.05 (**Figure 8A**). The heat map was used to compare the relative abundances of 34 differential lipids (**Figure 8B**). These outcomes indicated that QHD treatment could particularly impact the lipid in NAFLD rats. To explore the metabolic enrichment of the 34 differential lipids based on the KEGG database, we performed pathway analysis using MetaboAnalyst 5.0. As shown in **Figure 8D**, the differential lipids were enriched in glycerophospholipid metabolism.

### 3.5 Associations between differential gut microbiota and circulating lipid metabolites in NAFLD rats

The relationship between lipids, differential genera and biological parameters was assessed using Spearman’s correlation analysis (**Figure 8C**). When evaluating the correlations with changes in biological parameters, *Blautia* and *Lachnoclostridium* were positively correlated with liver weight, hepatic TG, and NAS score. *Bacteroides* was positively correlated with liver weight; *Clostridium_sensu_stricto_1* was negatively correlated with insulin, HOMA-IR, serum ALT, liver weight, hepatic TG, NAS score, and body weight; and *Turicibacter* was negatively correlated with FBG, serum ALT, liver weight, hepatic TG, and body weight.

The correlation analysis of 34 differential lipids and biological outcomes showed that 33 lipids were positively correlated with liver weight; 32 lipids were positively correlated with NAS score and body weight; 31 lipids were positively correlated with insulin, HOMA-IR, and hepatic TG; 30 lipids were were positively correlated with FBG, and 12 lipids were positively correlated with serum ATL. Nevertheless, PC (36:5) showed negatively correlated with FBG, HOMA-IR, and serum ATL.

Furthermore, we found that *Bacteroides* was positively correlated with 17 lipids; *Blautia* and *Lachnoclostridium* were positively correlated with 33 lipids; *Lactobacillus was* positively correlated with PE(16:0/18:2); and *Sporosarcina* was positively correlated with PC(36:5). Whereas *Clostridium_sensu_stricto_1* was negatively correlated with 32 lipids; and *Turicibacter* was negatively correlated with 18 lipids.

## 4 Discussion

The increasing risk of NAFLD has been confirmed by many studies. A recent global epidemiologic study of NAFLD found that the global prevalence of NAFLD is about 30% and rising (32). Individuals are increasingly being affected by NAFLD at an earlier age, meaning there is more time for them to develop severe complications (33). QHD has been clinically used for NAFLD treatment in China for decades. In recent studies, the mechanism of QHD in NAFLD is related to the biological functions of reducing oxidative stress, regulating intestinal microbiota, regulating body metabolism and signaling pathways, promoting browning of white adipose tissue, and inhibiting lipogenesis (9, 12, 14, 15, 34, 35). Furthermore, it has been reported that many bioactive components of QHD have significant anti-NAFLD activities. For example, the therapeutic effect of geniposide and chlorogenic acid combination on NAFLD was confirmed by ameliorating HFD-induced NASH in mice (36). In addition, Wan et al. demonstrated that chlorogenic acid may also prevent fatty liver by upregulating the expression of peroxisomal proliferator-activated receptor α (37). Scoparone is a potent constituent of “Yinchen” and a potential drug candidate for NAFLD, which has a broad spectrum of pharmacological activities, including anti-fibrotic, antioxidant, anti-inflammatory, and hypolipidemic properties (38). One of the active ingredients in QHD, emodin, an anthraquinone derivative and the active ingredient in Huzhang”, has been found to have a broad spectrum of pharmacological activities, such as anti-inflammatory, antiviral, antibacterial, anti-fibrotic, and hypolipidemic properties, and hepatoprotective activities (39). Curcumin is extracted from “Jianghuang” and has been shown to have several potentially therapeutic properties, including anti-inflammatory, antioxidant, and anti-fibrotic (40).

Understanding the functions connected with the microbial community is important because gut microbiota changes are related to NAFLD and the treatments (41). We herein proved that HFD caused dramatic decrease in bacterial diversity and richness, as verified by the reduction in richness, ace, and Chao1 indexes, whereas QHD administration significantly recovered the indexes. The gut microbiota composition in NAFLD rats was different from the other two groups according to β-diversity. LefSe was performed to identify genera of *Allobaculum, Bacteroides, Blautia, Clostridium_sensu_stricto_1, Lachnoclostridium, Lactobacillus, Romboutsia, Sporosarcina*, *Turicibacter*, and *Psychrobacter* as important microbial biomarkers. In our study, *Blautia* was found positively correlated with liver weight, hepatic TG and NAS score; and *Bacteroides* was positively correlated with liver weight. As mentioned in the literature, *Blautia* may be involved in alleviating obesity, inflammation and insulin resistance (42). What’s more, the most consistent gut microbiota signatures associated with NAFLD are increased *Bacteroides* (43). *Bacteroides* was the dominant genus in NAFLD rats, while the abundance of *Bacteroides* decreased after QHD administration in our study. It has been reported that *Bacteroides* and *Blautia* can produce acetate, which can negatively regulate insulin signaling in adipocytes, leading to suppressed fat deposition (44). However, previous studies found inconsistent results for *Blautia* (29). *Lachnoclostridium*, a short-chain fatty acids producer, was previously reported to be enriched and significantly downregulated by QHD (16), which was in line with our results. *Lachnoclostridium* was also positively correlated with NAFLD status, namely liver weight, hepatic TG, and NAS score in our study. Our study showed that *Clostridium_sensu_stricto_1* was significantly reduced by an 8-week HFD and was restored by QHD treatment. *Clostridium_sensu_stricto_1* was negatively correlated with insulin, HOMA-IR, serum ALT, liver weight, hepatic TG, NAS score, and body weight. Previous studies found that growth differentiation factor 15 regulates TG metabolism to modulate inflammation and showed a significant positive correlation with *Clostridium_sensu_stricto_1* (45). In our research, *Turicibacter* was negatively correlated with FBG, serum ALT, liver weight, hepatic TG, and body weight. *Turicibacter* showed a strong positive association with liver malondialdehyde in a previous study (46). In addition, gut dysbiosis in NAFLD is characterized by alterations in the intestinal barrier that allow bacteria or bacterial products, such as lipopolysaccharide, to enter the portal circulation. Bacterial translocation has been shown to increase the expression of specific receptors on the surface of hepatocytes, such as TLRs, which are relevant for a pro-inflammatory response mediated by IL-1β, TNF-α and interferons. It was found that lipopolysaccharide levels may be negatively correlated with *Allobaculum* (47). In addition, *Lactobacillus* has been reported to be associated with insulin resistance and correlated with FBG and glycated hemoglobin levels (48). While recent studies have identified gut microbiota associated with the potentially beneficial genera, including *Lactobacillus* (49). The level of *Lactobacillus* was higher in NAFLD rats than in the other groups in our study. However, whether the differential microbiota plays a corresponding role needs further verification.

Currently, biopsy is required in NAFLD patients to obtain a conclusive diagnosis, and screening for serum lipid differences is undoubtedly a promising method. As traditional lipid risk factors, TC and TG could not explicitly explain the pathogenic mechanism of NAFLD. Lipidomics can explore the structures and functions of the full range of lipids in an organism to explain the pathways and interactions, helping us to understand the complexity of lipid dysregulation in NAFLD (50, 51). HFD injures liver, thereby affecting lipid transport and metabolism. As a risk factor for the progression of NAFLD, lipids changed significantly. Thus, we investigated the circulating lipids in NAFLD rats treated with or without QHD, and explored whether lipids measured in serum could be correlated with gut microbiota in NAFLD. We identified signatures of serum lipid species in the rats. Untargeted lipidomics in this study revealed a total of 24 different lipid subclasses in both the negative and positive ion modes. Serum lipidomics analysis revealed that DG, TG, PA, PC, PE, PS, LPI and PG were mainly altered in NAFLD. We observed that DG, TG, PA, LPC, LPE and PAF levels were significantly reduced by QHD. We also found 34 NAFLD-related lipids that were regulated by QHD treatment. Among the 34 differential lipids, 33 lipids were positively correlated with liver weight; 32 lipids were positively correlated with body weight and NAS score; 31 lipids were positively correlated with insulin, HOMA-IR, and hepatic TG; 30 lipids were were positively correlated with FBG; and 12 were positively correlated with serum ATL, whereas PC (36:5) was negatively correlated with serum ATL, FBG and HOMA-IR. As one of the primary structural lipids of eukaryote cell membranes, glycerophospholipids regulate multiple cellular metabolic processes (52). PE and PC are the most abundant phospholipids in all mammalian cell membranes. In our study, among the 34 lipids, PE lipid molecules were decreased significantly in the NAFLD+QHD group. The hepatic PC/PE molar ratio is a key determinant of liver health. Changes in the hepatic PC/PE molar ratio have been associated with the development of NAFLD in humans (53). In our experiment, NAFLD rats had significantly lower serum PC/PE levels, which were elevated after QHD administration compared to the model group, although there was no statistical difference (**Figure 7F**). Bioactive lipid intermediates such as PC, LPC, are proposed to be associated with the development of hepatic lipotoxicity or insulin resistance, which are important players in NAFLD (54). In the liver, PC is involved in the secretion of very-low-density lipoprotein and the metabolism of high-density lipoprotein. Synthesized PC is the most abundant lipid component (up to 70% molar ratio) of plasma very-low-density lipoprotein and also constitutes nearly 40% of nascent high-density lipoprotein (55). In this study, QHD treatment increased serum PC (38:4) and PC (36:5), and decreased PC (17:4/18:1) and PC (17:4/18:0). LPC is derived by hydrolytic cleavage of PC catalyzed by the phospholipase A2 enzymes (54). In the liver, LPC upregulates genes involved in cholesterol biosynthesis and downregulates genes involved in fatty acid oxidation. Higher concentrations of LPC can disrupt mitochondrial integrity and increase cytochrome C release (55). The essential signaling molecules LPE and LPC are also derived from PE and PC, respectively. In our study, LPC and LPE subclasses were decreased in the NAFLD+QHD rats. Interestingly, PA was decreased in the NAFLD+QHD rats. PA can be converted into cytidine diphosphate-diacylglycerol, a substrate for the synthesis of PI, PG, and cardiolipins, or can be dephosphorylated by phosphatidate phosphohydrolase to form DG, which acts as a precursor molecule for the synthesis of TG, PC, PE, and PS. Diacylglycerol acyltransferase catalyzes DG acylation, the last step in TG synthesis. The newly synthesized TG can be directed from the endoplasmic reticulum lipid bilayer to form cytosolic lipid droplets (56, 57). NAFLD begins with the accumulation of TG in the liver (20). In our study, both DG and TG were increased in rats with NAFLD, indicating increased accumulation of lipid droplets. However, reduced levels of DG and TG were observed in NAFLD+QHD rats. Cer is the key precursor in the biosynthesis of various sphingolipids. Cer and SM are incorporated into VLDL during lipoprotein assembly by microsomal TG transfer protein. Phospholipid transfer protein can transfer SM between VLDL and HDL (58). Through oxidization, the FAs taken up by hepatocytes either produce energy and ketone bodies, or are re-esterified to DGs and TGs. Newly-synthesized TG can be secreted as VLDL or be stored as lipid droplets (59). However, compared to the NAFLD group, SM and Cer did not change in the NAFLD+QHD group. Our results indicate that QHD appears to be promising for alleviating lipid metabolic disorders in rats with HFD-induced NAFLD, and provide the basis for similar studies in larger cohorts to identify new lipid molecular species that have not been associated with NAFLD previously.

Our focus was then on the relationship between gut microbiota and lipid metabolism. The gut microbiome has been shown to influence lipid metabolism and lipid levels in peripheral tissues. A recent meta-analysis found that probiotics can effectively affect blood lipids (60). A multi-omics study showed that microbiota can induce monounsaturated fatty acid production and polyunsaturated fatty acid elongation, resulting in significant changes in the acyl chain spectrum of glycerol phospholipids (61). Changes in gut microbiota composition that occur during NAFLD development can interfere with lipid metabolism in the liver, allowing for fat accumulation and lipotoxicity (20). An altered gut microbiota can alter the synthesis of fasting-induced adipocyte factor, a lipoprotein lipase inhibitor involved in the release of free FAs from VLDL particles into the liver. Therefore, suppression of this factor is associated with an increased lipid accumulation in the liver (22). Several studies have suggested an association between lipid metabolism and gut microbiota (51, 62–64). New evidence in our study showed that gut microbiota composition was associated with lipid profiles after QHD treatment. It was found that the gut microbiota in our study was largely involved in lipid metabolism according to the pathway analysis based on the KEGG database (**Figure 4C**), in addition, the levels of several lipid metabolites were associated with the different genera of gut microbiota levels, revealing a potential communication of gut microbiota with plasma lipid profiles in NAFLD. The comprehensive analysis of lipidomics and 16S rRNA amplicon sequencing highlighted a possible microbiota-derived biosynthesis of lipids. Furthermore, our study revealed that the lipid metabolic profile in rat serum was highly correlated with *Blautia*, *Lachnoclostridium, Clostridium_sensu_stricto_1* and *Turicibacter*. Based on these results, a potential link between gut microbiota and serum lipid profiles was suggested. However, it is noteworthy that genetics, gut microbiota, and dietary intake may also contribute to host lipid profiles. Moreover, the underlying mechanisms of these associations remain unclear and require further investigation.

However, the limitation of the study was that the supplementation with specific species of bacteria or lipid metabolites needed to be validated for NAFLD rats. To clarify the relationship between lipids and gut microbiota in NAFLD models, further in-depth research is needed. Moreover, studies in human subjects with or without NAFLD have yet to be performed.

## 5 Conclusion

QHD supplementation can systematically alleviate hepatic steatosis in NAFLD rats. The application of a multiomics approach reveals that QHD supplementation can improve the structure of the dysfunctional gut microbiota and regulate DG, TG, PA, LPC, LPE and PAF. The differential circulating lipids were mainly associated with genera *Bacteroides, Blautia, Lachnoclostridium, Clostridium_sensu_stricto_1*, and *Turicibacter*, which were significantly correlated with biological parameters. Taken together, these findings indicate that the regulation of gut microbiota and lipid homeostasis may be critical in the mechanisms of QHD in the treatment of NAFLD, providing a scientific basis for future clinical applications and experimental studies of QHD.

## Data availability statement

The original contributions presented in the study are publicly available. This data can be found here: https://www.ncbi.nlm.nih.gov/, BioProject: PRJNA951499.

## Ethics statement

The animal study was approved by The Animal Studies Ethics Committee of Shanghai University of Traditional Chinese Medicine. The study was conducted in accordance with the local legislation and institutional requirements.

## Author contributions

YN: Writing – original draft, Writing – review & editing. XW: Investigation, Writing – original draft. QW: Methodology, Writing – review & editing. YY: Investigation, Writing – original draft. YX: Writing – original draft. YL: Methodology, Writing – original draft. QF: Writing – review & editing. MZ: Writing – review & editing. XG: Writing – original draft, Writing – review & editing.
